# Linking Data on Nonfatal Firearm Injuries in Youths to Assess Disease Burden

**DOI:** 10.1001/jamanetworkopen.2024.36640

**Published:** 2024-09-30

**Authors:** Lauren A. Magee, Damaris Ortiz, Zachary W. Adams, Jodi L. Raymond, Brigid R. Marriott, Matthew P. Landman, Joseph O’Neill, Tiffany L. Davis, Jamie Williams, Kendale Adams, Jessica Belchos, J. Dennis Fortenberry, Peter C. Jenkins, Megan L. Ranney

**Affiliations:** 1 Paul H. O’Neill School of Public and Environmental Affairs, Indiana University Indianapolis, Indianapolis; 2 Department of Surgery, Indiana University School of Medicine, Indianapolis; 3 Smith Level 1 Shock Trauma Center, Sidney & Lois Eskenazi Hospital, Indianapolis, Indiana; 4 Adolescent Behavior Health Research Program, Indiana University School of Medicine, Indianapolis; 5 Riley Hospital for Children at Indiana University Health, Indianapolis; 6 Department of Pediatrics, Indiana University School of Medicine, Indianapolis; 7 Indiana University Health Methodist Hospital, Indianapolis; 8 Ascension St Vincent Hospital–Indianapolis, Indianapolis, Indiana; 9 Indianapolis Metropolitan Police Department, Indianapolis, Indiana; 10 Division of Adolescent Medicine, Department of Pediatrics, Indiana University School of Medicine, Indianapolis; 11 Yale School of Public Health, Yale University, New Haven, Connecticut

## Abstract

This cross-sectional study estimates the incidence of nonfatal firearm injuries among children and young adults after linking patient-level police and trauma registry data.

## Introduction

Firearm injury is a public health crisis in the US, especially for children and young adults.^1^ Yet, the true burden of nonfatal firearm injuries is unknown; existing national data, usually obtained from hospitals, are incomplete.^2,3^ Trauma registry (TR) data may be easier to access than full hospital health record data. In this study, we assessed the impact and characteristics of linking patient-level police and TR data to estimate the incidence of nonfatal firearm injuries (NFIs) among children and young adults.

## Methods

We performed a cross-sectional, descriptive analysis of NFIs among people aged 0 to 27 years in Indianapolis, Indiana, between January 1, 2016, and December 31, 2021. We matched individual-level police data from the Indianapolis Metropolitan Police Department Nonfatal Shooting Database with TR data from all 4 level 1 trauma centers in the city (3 adult, 1 pediatric). Nonfatal firearm injuries are defined as a penetrating injury caused by a projectile weapon with a powder discharge. Data use agreements were established to maintain privacy and ethical safeguards. This study was approved by the Indiana University Institutional Review Board, which also waived informed consent because we used secondary data from TR and police records. We followed the STROBE reporting guideline.

We matched records using police data and TR data based on survivor’s last name, first name, and event date (exact matches and probabilistic matches at 0.9 threshold).^4^ Ten percent of the records were manually reviewed to ensure accuracy of matches; the remaining nonmatched records were confirmed manually.

After incidents were classified as matched or unmatched, we performed χ^2^ tests to examine differences based on demographic and injury characteristics. Race and ethnicity were included because of disparities in firearm violence exposure and categorized by self-reported administrative data. Analyses were performed using Stata, version 18 (StataCorp LLC), and 2-sided *P* < .05 indicated statistical significance.

## Results

We identified 2242 unique child and young adult survivors injured in 2345 NFI events (includes repeat survivors); of these events, 1960 (83.6%) involved males and the mean (SD) survivor age was 20.6 (4.2) years. The injuries included 2228 from police data and 1680 from TR data (Figure). Overall, 1563 NFIs (66.7%) matched between police and TR datasets, of which 1227 records matched, 224 additional records were hand matched across systems, and 112 matched in the Records Management System, which includes all events reported to police that are not in the Indianapolis Metropolitan Police Department Nonfatal Shooting Database. Among 782 nonmatched records, 665 (85.0%) were identified only in police data; 117 (15.0%), only in TR data.

**Figure.**
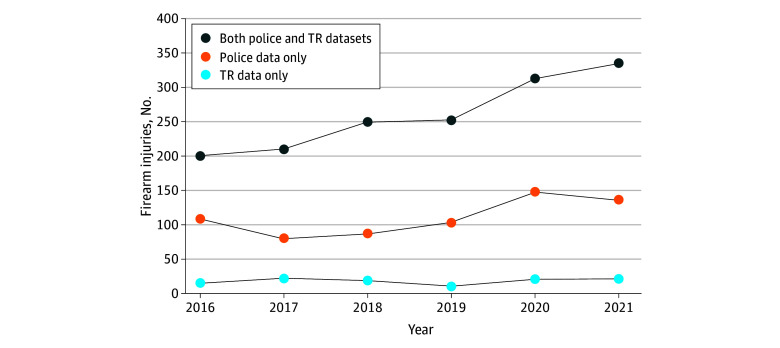
Annual Incidence of Nonfatal Firearm Injuries Identified by Police and Trauma Registry (TR) Data, Indianapolis, Indiana, 2016 Through 2021

Linking police and TR data significantly increased the estimated incidence of NFIs among Black survivors among both matched (1286 records [82.3%]) and unmatched records (police, 515 [77.4%]; TR, 75 [64.1%]) (*P* < .001) (Table). Of unmatched records, TR records identified the most Hispanic survivors (15 [12.8%]) and White survivors (22 [18.8%]) (*P* < .001). Assault-related shootings (1298 [83.0%]) were the injury intention most often identified in both systems.

**Table.** Match Results for Police and TR Data by Survivor Demographic and Injury Characteristics, Aged 0 to 27 Years, Indianapolis, Indiana, 2016 Through 2021^a^

Characteristic	NFI events, No. (%) (N = 2345)^b^			*P* value
	Both systems (n = 1563)	Police only (n = 665)	TR only (n = 117)	
**Survivor**				
Race and ethnicity				
Black	1286 (82.3)	515 (77.4)	75 (64.1)	<.001
Hispanic	23 (1.5)	35 (5.3)	15 (12.8)	
White	250 (16.0)	112 (16.8)	22 (18.8)	
Unknown	4 (0.3)	3 (0.5)	5 (4.3)	
Sex				
Male	1299 (83.1)	562 (84.5)	99 (84.6)	.64
Female	264 (16.9)	102 (15.3)	18 (15.4)	
Unknown	0	1 (0.2)	0	
Age group, y				
<12	40 (2.6)	13 (2.0)	3 (2.6)	.65
12-17	243 (15.5)	114 (17.1)	24 (20.5)	
18-22	743 (47.5)	318 (47.8)	48 (41.0)	
23-27	537 (34.4)	220 (33.1)	42 (35.9)	
**Injury**				
Intent				
Assault	1298 (83.0)	544 (81.8)	89 (76.1)	<.001
Unintentional	171 (10.9)	105 (15.8)	10 (8.6)	
Self-inflicted	25 (1.6)	8 (1.2)	2 (1.7)	
Self-defense	47 (3.0)	8 (1.2)	NA	
Undetermined	22 (1.4)	NA	16 (13.7)	
Motive or circumstances				
Interpersonal	326 (20.9)	147 (22.1)	NA	.001
Illegal activity	223 (14.3)	82 (12.3)	NA	
Bystander	17 (1.1)	28 (4.2)	NA	
Other or unknown	851 (54.4)	295 (44.4)	NA	
Unintentional	146 (9.3)	113 (17.0)	NA	

Abbreviations: NA, not available; NFI, nonfatal firearm injury; TR, trauma registry.

^a^ Survivor demographic characteristics and injury intent were defined by police records for matched records and original data source for unmatched records. Race and ethnicity are included due to disparities in firearm violence exposure. Event motive and circumstances were defined by police records.

^b^ Because of rounding, percentages may not sum to 100%.

Among 1563 matched records, discrepancies in survivor race and ethnicity (111 [7.1%]) and injury intent (325 [20.8%]) existed within systems. For 236 cases (15.1%), police identified assault or unintentional injury while the TR indicated the intent as undetermined, and for 72 cases (4.6%), police data indicated self-defense or an unintentional shooting, whereas the TR indicated assault (*P* < .001).

## Discussion

In this cross-sectional study, the estimated disease burden of child and young adult NFIs increased substantially when TR and police data were linked. Variability in case descriptions—including race and ethnicity and intent—existed when comparing systems. These results suggest a high likelihood of missing cases should the datasets not be joined, particularly for Black and Hispanic survivors. Study limitations include missing data on firearm injuries that were not reported to police and for which medical treatment was not sought at the included trauma centers. This study’s findings demonstrate the feasibility and necessity of collaborating across health systems and police departments to improve case definition and to combine data across TR and police systems for violence prevention and evaluation initiatives.^5,6^

## Notes

Supplement.
Data Sharing Statement
