# Supplementary material for: Linking Data on Nonfatal Firearm Injuries in Youths to Assess Disease Burden
Source: JAMA Netw Open. 2024 Sep 30;7(9):e2436640. doi: 10.1001/jamanetworkopen.2024.36640 (PMC11443348; doi:10.1001/jamanetworkopen.2024.36640)
Supplement: Supplement. — Data Sharing Statement [file jamanetwopen-e2436640-s001.pdf]

Data Sharing Statement

Magee. Linking Data on Nonfatal Firearm Injuries in Youths to Assess Disease Burden. *JAMA Netw Open*. Published September 30, 2024. doi:10.1001/jamanetworkopen.2024.36640

Data
Data available: No

Additional Information
Explanation for why data not available: HIPPA protection and law enforcement sensitive.
